# Acute effects of mental recovery strategies in simulated air rifle competitions

**DOI:** 10.3389/fspor.2023.1087995

**Published:** 2023-05-15

**Authors:** Fabian Loch, Alexander Ferrauti, Tim Meyer, Mark Pfeiffer, Michael Kellmann

**Affiliations:** ^1^ Department of Sport Psychology, Faculty of Sport Science, Ruhr University Bochum, Bochum, Germany; ^2^ Department of Training and Exercise Science, Faculty of Sport Science, Ruhr University Bochum, Bochum, Germany; ^3^ Institute of Sports and Preventive Medicine, Saarland University, Saarbrücken, Germany; ^4^ Department of Theory and Practice of Sports, Institute of Sport Science, Mainz, Germany; ^5^ School of Human Movement and Nutrition Science, The University of Queensland, Brisbane, Australia

**Keywords:** fatigue, psychological, performance, rest, mental break

## Abstract

**Objectives:**

The present study aimed to assess the perception and change of mental and physical fatigue and to examine acute effects of mental recovery strategies in air rifle athletes across simulated competition days with two consecutive competition bouts.

**Design:**

We conducted a randomized counterbalanced crossover study.

**Method:**

22 development air rifle athletes (M_age_ = 17.77 ± 4.0) of a regional squad participate in the study. The Short Recovery and Stress Scale (SRSS), perception of mental fatigue, physical fatigue, concentration and motivation as well as differential Ratings of Perceived Exertion (RPE) were used to assess recovery-stress states and fatigue states. During a recovery break, participants underwent two mental recovery strategies (powernap, systematic breathing) or a control condition. Total shooting scores were recorded for both competition bouts.

**Results:**

Study results revealed a significant increase of post ratings for mental (*p* < .001) and physical fatigue (*p* < .001) for both competition bouts. The correlation coefficient between change in mental and physical fatigue for both competitions revealed a shared variance of 7.9% and 18.6%, respectively. No significant group-based acute effects of the use of mental recovery strategies on shooting performance, and psychological and perceptual measures were found. On an individual level, results illustrated statistical relevant improvements of shooting performance after powernapping or systematic breathing.

**Conclusion:**

Mental and physical fatigue increased and accumulated across a simulated air rifle competition and mental fatigue emerged as a separate construct from physical fatigue. The use of strategies to accelerate mental recovery on an individual level (e.g., powernap, systematic breathing) may be a first step to manage a state of mental fatigue, but further studies on mental recovery strategies in an applied setting are needed.

## Introduction

Athletes often faced with sport-specific multidimensional stressors comprising a significant amount of physical and mental stress before, during, and after competitions, which in turn results in physical as well as mental fatigue (1, 2) and appears to have an impact on the balance of individual recovery-stress states (3). There is a special focus on the characteristics of sports with multiple intensive competition bouts (e.g., qualification heat and final, distinct contest) in single day, which leads to physical (e.g., specific physiological and technical requirements) and mental demands (e.g., sustained concentration, attentional focus, control) resulting in an acute state of mental fatigue (3, 4).

Mental fatigue is defined as a psychobiological state caused by demanding cognitive activity and associated with low levels of energy and feelings of tiredness (5, 6). Impacts of a mental fatigue state can be seen in impairments of executive functions (e.g., inhibition, working memory and mental flexibility) as well as of decision-making performance. Regarding this, prolonged mental exertion negatively influences attention, action monitoring, and cognitive control, which can result in a lack of concentration and alertness (7). Thus, activities that require attention may cause mental fatigue, that can lead to impairments in executive functions as well as cognitive abilities. Applied to the air rifle setting, cognitive demanding conditions before competitions such as the use of smartphones (e.g., social networks, playing video games), handling of dual tasks during pre-competition preparation, detailed briefings of tactical and technical strategies or over-analysis of preceding competitions may cause a state of mental fatigue (8).

It has been established that mental fatigue has a negative impact on physical performance, which has been attributed to an increased perception of effort (9, 10). Moreover, current research has revealed that mental fatigue negatively impacts physical, technical, and tactical skills (11, 12). In addition, a combination of subjective, behavioural, and physiological manifestations has been used to identify mental fatigue.

Regarding the study of Van Cutsem et al. (13), on how mental fatigue impairs human performance with the focus on how the execution of one cognitive task can impact the performance on a subsequent task, the aim was to analyze all possible measures that could be related to a mental fatigue-associated drop in performance. As a holistic approach, systemic-related measures of both central (e.g., functional magnetic resonance imaging) and peripheral (e.g., breathing rate, heart rate variability) neurophysiology, subjective measures (e.g., mental fatigue, motivation) and behavioural measures (e.g., evaluating cognitive performance) were included in the study. In addition, findings of Russell et al. (14), underline that key outcomes appear to be primarily on the subjective and behavioural level. Outcomes on a subjective level are mainly feelings of tiredness, lack of energy, decreased motivation, and alertness, whereas on the behavioural level a decline in accuracy and/or reaction time responses in cognitive tasks are linked to mental fatigue (15).

As a countermeasure the concept of mental recovery is attracting increasing attention in sport-scientific research (16, 17). Mental recovery aims at obtaining baseline levels of mental abilities (e.g., concentration, vigilance, attention) and the restoration of mental energy (16, 18). Hence, it includes to refuel required resources, recharge on a mental level, rest the mind in order to reduce upcoming stress, and deal with disturbing thoughts (19).

In the psychophysiological process of recovery, resources play an essential role, which can be divided into physical, mental and energetic components (20). Resources are related to the energetic level of a person which is dependent on the arousal level. In the context of refuel resources, energy or the energetic level of a person is a main factor, which includes both physical energy (e.g., the capacity to perform) and mental activation (e.g., subjective feeling of being energized). Moreover, additional factors such as motivation and concentration appear to be relevant for the process of resource production, protection, and depletion (21). Therefore, restoring energy resources is an essential aspect of (mental) recovery. The idea of mental recovery also underlines the crucial role of self-regulation in the process of mental recovery in relation to the finding and the implementation of the best and adequate recovery for oneself (1, 22). The regulation of thoughts, feelings, and emotions is essential to recover on a mental level which is necessary to compete in the previous competition bout. Thus, the process of mental recovery coincides with the idea of recovery self-regulation which can be considered as the process of moving from an actual state (e.g., high mental fatigue, high stress) to a preferred or required future state (e.g., optimal state of recovery) of physical as well as mental activation and readiness by minimizing the discrepancy between both states (1). Therefore, to achieve a state of mental recovery, mental breaks are essential for athletes to be able to physically and mentally “switch off” from sport-related demands during the recovery phase (23).

Overall, it can be assumed that the specific characteristics of all-day competitions with the alternating competitions periods and fixed rest periods are mentally demanding and can lead to an acute state of mental fatigue (3). The specific competition structure highlights the psychologically demanding conditions for the athletes and strengthens the importance of adequate and appropriate competition recovery, i.e., the promotion of a mental recovery state as a countermeasure against mental fatigue (1, 17).

Air rifle competitions consist of two or even three single competition bouts (i.e., qualification round, eliminations round, final). During each competition, athletes have to shoot 60 shots on a stationary target in 10-meter distance from a standing shooting position within 75 min (24). As professional shooting tournaments often last several hours (25), air rifle athletes have to stay alert and sustain concentration over the course of the competition day and must not lose their competition tension (26). In air-rifle shooting, technical, physical, and psychological determinants are crucial factors for an optimal shooting performance, which athletes have to retrieve in high-pressure situations (27). There are only a few air rifle studies, predominantly with a focus on specific shooting technique parameters (e.g., stability of hold, postural balance, aiming accuracy, and trigger control) and their relation to shooting performance (28). In addition to physical determinants (e.g., sense of balance, rhythm, reactivity, endurance, strength), psychological determinants including concentration capacity, attentional focus, and impulse control can be described as key factors and are essential for the attainment of peak performance in sports (29).

So far, the influence of mental fatigue on repeated competition bouts of high efforts over extended periods in a realistic or a natural competitive sport setting is yet to be investigated (30). Furthermore, little is known about the use and beneficial effects of mental recovery strategies in an applied sport setting (31). The scoping review of Loch et al. (17) summarizes the current knowledge of mental recovery with the focus on a more short-term nature of rest periods (e.g., short periods between competition bouts on a single day) and discusses possible mental recovery strategies for the use in sports. Based on literature, several strategies such as powernap and systematic breathing appear to have positive effects on mental states such as concentration, attention, and cognitive function as well as on performance outcomes (32, 33).

In their laboratory-based randomized cross-over study, Loch et al. (18), examine acute effects of mental recovery strategies on subjective-psychological measures and on cognitive performance after a mentally fatiguing task with undergraduate and graduate students. Regarding acute effects of mental recovery strategies, results revealed significant time effects for stress and recovery states, fatigue states, and cognitive performance outcomes but findings could not reveal positive effects of powernap or systematic breathing in mental recovery. The purpose of the present study was to adapt these findings and transfer them to a more sport-related and applied setting (e.g., selection of participants, familiar training environment, simulated competition setting) to clarify that these strategies could be suitable for the use as mental recovery strategies especially in short rest periods in sports (17). Thus, the novelty of the present study is, that we pursue two aims to gain new insights into the effects of mental breaks in a more applied setting. First, we want to assess the perception and the change of mental and physical fatigue in air rifle athletes over the course of a simulated competition day. Second, we want to examine acute effects of the use of mental recovery strategies in an implemented recovery break on shooting performance (i.e., total shooting score) and psychological (e.g., recovery-stress states, progress of fatigue states, perceived exertion) measures. Thus, we hypothesized (1) that the progression of two air rifle competitions lead to an increase in stress states and the perception of fatigue states as well as to a decrease in recovery states in air rifle athletes and (2) that the use of mental recovery strategies leads to counteractive development as well as to an improvement of the shooting performance.

## Material and methods

### Participants

The study was conducted with a total of 22 development air rifle athletes (13 female, 9 male) between 14 and 28 years (*M*_age_ = 17.77 ± 4.0; *M*_height_ = 172.2 ± 7.5 cm; *M*_weight_ = 66.2 ± 9.1 kg; training per week: *M* = 5.64 h ± 1.81) of a regional squad of national performance level. In the course of collection of demographic data, we asked participants for the frequency of the application of recovery strategies in general as well as the specific use in training and competition using a 5-point Likert scale (0–4) ranging from “never” to “always”. The experience with recovery strategies of the participants lies within a small to medium range and participants rarely use recovery strategies during training and competition. Participants were verbally informed about the course of the study and provided written informed consent. The study was approved by the local ethics committee.

### Procedure & measures

**Figure 1:**
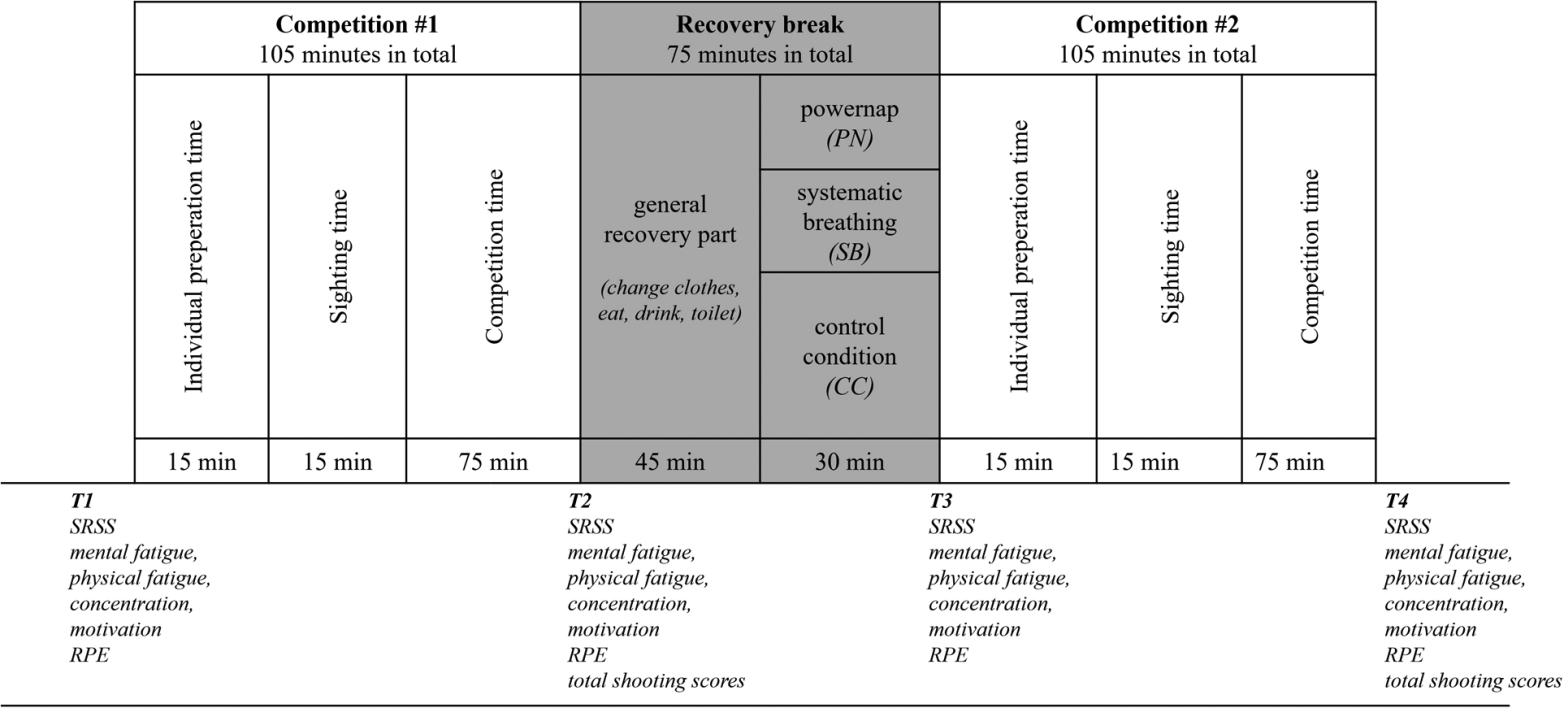
Schematic overview of the study design. SRSS = Short Recovery and Stress Scale, RPE = Differential Ratings of Perceived Exertion.

A randomized counterbalanced crossover study with three separated test days was conducted. During a single test day, athletes had to complete two consecutive simulated competitions with equal structure based on the official ISSF air rifle competition rules (i.e., 60 shots in a maximum of 75 min) separated by a recovery break of 75 min (24). Participants' *total shooting scores* for both simulated competitions (#1, #2) were assessed using the International Sport Shooting Federation's qualifying competition procedures for air rifle events using electronic targets (24). Athletes were required to fire their weapons in standing positions from a 10-meter distance to the target in an indoor shooting range. In total, the completion of a single simulated competition day results in an overall duration of 4:45 h (Figure 1). The time structure was maintained for all three test days.

In the study, the course of the first competition bout (#1) consisted of three different parts including individual preparation time (15 min), air rifle sighting time (15 min), and competition time (75 min). The recovery break comprised a general part including locking the gun, removing of individual rifle clothing, and the possibility to eat, drink, and use the toilet as well as a specific part including a period of mental recovery. During mental recovery (30 min prior to the second competition bout), participants underwent one of two mental recovery interventions (powernap [PN], systematic breathing [SB]), or a control condition [CC] for 20 min. Following the intervention, participants performed the second competition bout (#2). The individual order of intervention groups and control group was determined by using a random number generator. During the entire competition day, athletes were asked to answer the German paper pencil version of the Short Recovery and Stress Scale (SRSS), the perception of *mental fatigue*, *physical fatigue*, *concentration,* and *motivation* as well as differential Ratings of Perceived Exertion (RPE). The completion of the questionnaires was always done prior to a competition bout and once again at the end of the competition as well as prior to the start and at the end of the recovery break. Measurement points were defined as T1 (pre-competition 1), T2 (post-competition 1), T3 (pre-competition 2) and T4 (post-competition 2). Moreover, an evaluation of the mental recovery strategies was applied. The individual shooting scores were automatically recorded for both single competitions.

### Total shooting scores

The *total shooting scores* are defined as the overall shot score of 60 shots consisting of 6 series of 10 shots. In air rifle shooting the shot score ranges between 0 and 10.9. Individual scores were recorded on the electronic scoring system, Meyton Software (leading scoring system of the national German Sport Shooting Federation). Moreover, individual competition duration was collected.

### Recovery-stress states

The Short Recovery and Stress Scale (SRSS) measures the current recovery-stress state of an athlete multidimensionally with eight items on emotional, mental, physical, and overall level (34). The SRSS grouped into the Short Recovery Scale including four recovery-related items (*Physical Performance Capability*, *Mental Performance Capability*, *Emotional Balance*, and *Overall Recovery*) and the Short Stress Scale including four stress-related items (*Muscular Stress*, *Lack of Activation*, *Negative Emotional State*, and *Overall Stress*). For each item of the SRSS relevant adjectives served as descriptors (e.g., *Physical Performance Capability: strong, physically capable, energetic, full of power*) Answers were provided on a 7-point Likert Scale (0–6) ranging from “*does not apply at all”* to “*fully applies”*. The eight scores of the SRSS have shown acceptable internal consistencies, ranging from *α* = .70 to *α* = .76. The SRSS is validated and is available in a German and English version (34, 35).

### Fatigue states

To measure different fatigue states of the athletes four separate constructs were examined. *Mental fatigue*, *physical fatigue*, *concentration*, and *motivation* were rated using paper-based 100-mm Visual Analogue Scales (12, 36). The VAS have been reported as a valid and reliable instrument to measure *mental fatigue* (37) and are common scales in mental fatigue and physical performance literature (10, 30, 38, 39). Participants were asked to rate their current levels of *mental fatigue*, *physical fatigue*, *concentration*, and *motivation* by marking a single mark on a separate 100 mm horizontal line, ranging from “*none at all*” (0) to “*maximum”* (100). No other markings were displayed on the scales. Following the procedure of Russell et al. (30), we provided all participants short definitions of mental fatigue, physical fatigue, concentration and motivation to improve the metacognition of these constructs (e.g., *mental fatigue: “a psychobiological state caused by prolonged periods of cognitive activity”*).

### Ratings of perceived exertion

The perceived exertion was determined prior and after each competition bout using the CR-10 RPE scale (40). The RPE score (*RPE global*) represents a global rating of perceived exertion for the complete competition bout. In addition, adjusted versions of the RPE scale were used to provide a more detailed quantification of exertion on a physical (*RPE physical*) and on a mental level (*RPE mental*) (41, 42). Before the start of the study, all participants were familiarized with the CR-10 RPE scale.

### Mental recovery interventions

The selection of interventions was mainly based on the procedures and findings of previous studies (18, 43). The PN intervention consisted of an introduction period and a 20 min nap. All participants were instructed to nap on a training mat in a comfortable lying position. The SB intervention was composed of an introduction period, a main part, and a retrieval period. A breathing rhythm in which the exhalation phase was required to be twice as long as the inhalation period (leading to a 3s/6s or 4s/8s rhythms), was applied. All participants were instructed to lie on a training mat and were guided through a pre-recorded audio instruction. In the control condition participants had the possibility to read a collection of magazines of different topics (e.g., news magazines). During all three interventions, participants had no access to their mobile phones. Additional intervention instructions were verbally given by an educated researcher.

### Evaluation of the recovery break

A self-designed manipulation check was administered after each intervention session to gain insight into participants’ evaluations of the interventions (18). Participants were asked to evaluate the conducted mental recovery strategy relating to the efficacy (*As how effective did you experience the recovery method?*), the individual preference (*How did you like the recovery method?)*, and the feasibility (*I was able to apply the mental recovery method correctly)* of the current recovery intervention using a 7-Point Likert Scale (0–6) ranging from “*not at all*” to “*fully applies*” or from “*not at all implemented*” to “*perfectly implemented*”. On a qualitative level, participants had the chance to give positive or negative feedback regarding the used mental recovery strategies. Moreover, further information on additionally used methods was gathered.

### Statistical analyses

A power analysis was conducted using G*Power (parameters: repeated measures ANOVA, within-between interaction, *f* = .25, *p* = .05, power = .80, number of groups: 3, number of measurement points: 4) yielding a sample size of 30 participants. The sample calculation was based on the reported parameters of the study by Loch et al. (18). For the present study, 32 participants could be recruited, but due to the exclusion of 10 participants only 22 participants could be included in the analyses. Thus, an analysis a-posteriori revealed a power of .64.

Following normality testing, Wilcoxon-signed rank tests were used to assess pre-post changes in *mental fatigue* and *physical fatigue*, change in *mental fatigue* compared to change in *physical fatigue*, and between-competition differences. The acute change of *mental fatigue* and *physical fatigue* was calculated using the difference between values of *mental fatigue* and *physical fatigue* of #1 and #2. Spearman's rank correlational test determined the shared variance between acute changes in *mental fatigue* and *physical fatigue* and were used to establish the relationship between competition duration, TSS, RPE scores and acute changes of *mental fatigue* and *physical fatigue* (#1 and #2).

A multivariate analysis of repeated measurements was conducted using linear mixed models (lme4 package) in R software (26, 44). *Total shooting scores*, SRSS values, values of *mental fatigue*, *physical fatigue*, *concentration,* and *motivation* and RPE scores were defined and structured as dependent variables. In addition to measurement points (single measurement points as predictor variables), intervention group (predictor variable PN, SB, CC) as well as interactions of measurement points and respective intervention groups were defined as predictor variables. Predictor variables PN, SB, and CC were dummy-coded and assigned to respective measurement points depicting whether and when the intervention was implemented.

Modelling was implemented gradually, starting with intercept-only models to allow calculations of interclass-correlation coefficients (ICC). Next, predictor variable measurement point was added to the respective model, followed by intervention group and interaction of measurement point and intervention group. Models were fitted with Maximum-Likelihood estimation. To test for effects of mental recovery interventions and control condition on shooting performance (i.e., total shooting scores) during the simulated competition day, models with random intercepts and fixed slopes for predictor variables measurement point (MP) and intervention group or rather their interaction, were estimated [(TSS ∼ MP * Powernap + (1 | ID); (TSS ∼ MP * Systematic Breathing + (1 | ID); (TSS∼MP * Control Condition + (1 | ID)]. In addition, effects of mental recovery interventions and control condition on measures of recovery-stress states, fatigue states, and perceived exertion were examined. Specifically, models with random intercepts and fixed slopes for (predictors) measurement points and each intervention group, more specifically their interaction, were fitted [e.g., (SRSS ∼ MP * Powernap + (1 | ID); (VAS ∼ MP * Powernap + (1 | ID); (RPE ∼ MP * Powernap + (1 | ID)]. Alpha was set at 0.05. All data were presented as means ± SD.

## Results

**Figure 2:**
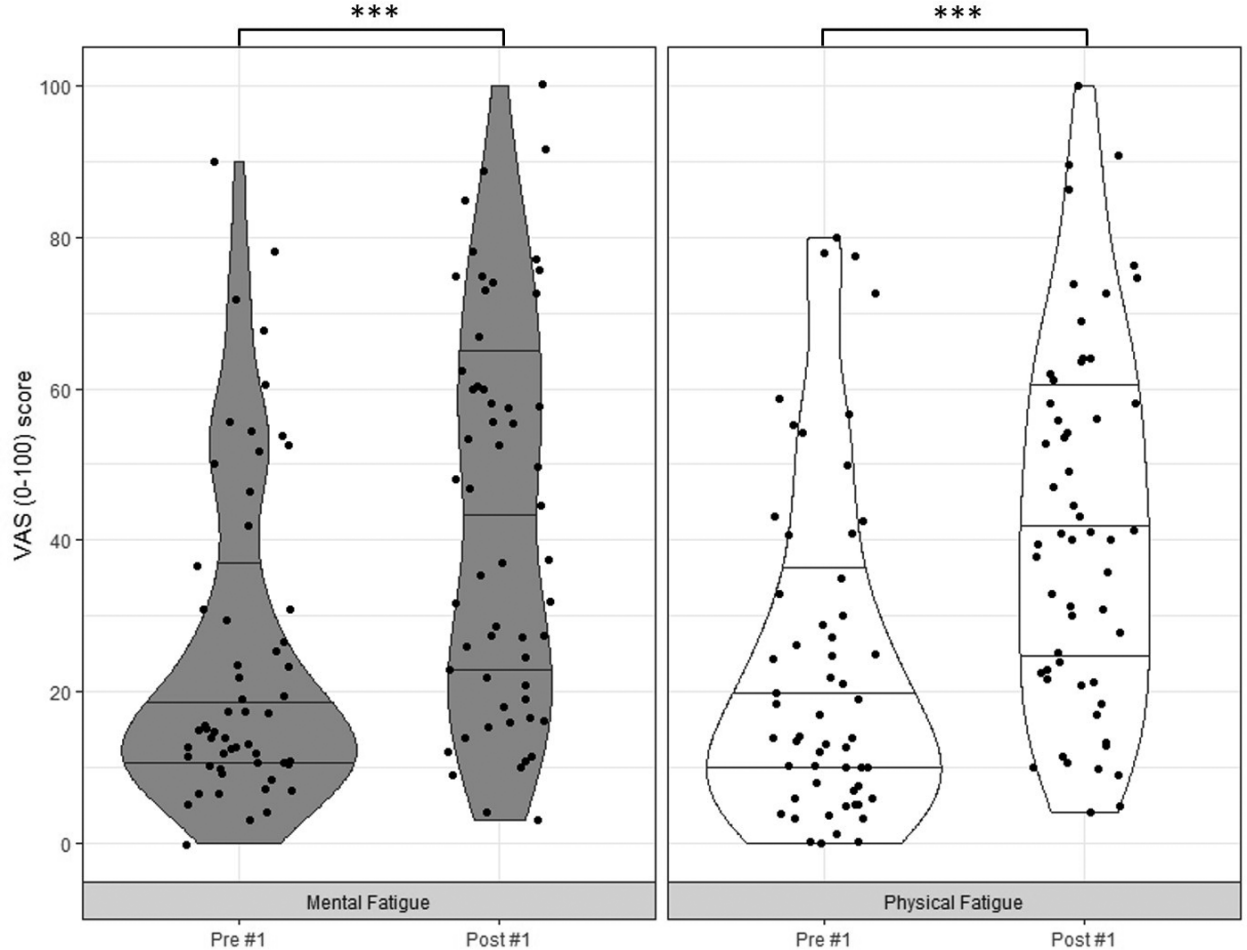
Violin Plots including data points for pre- and post-competition ratings of *mental fatigue* and *physical fatigue* for competition #1: minimum, Q1, median Q4 and maximum scores. Notes: VAS = Visual Analogue Scale. ***p < .001.

**Figure 3:**
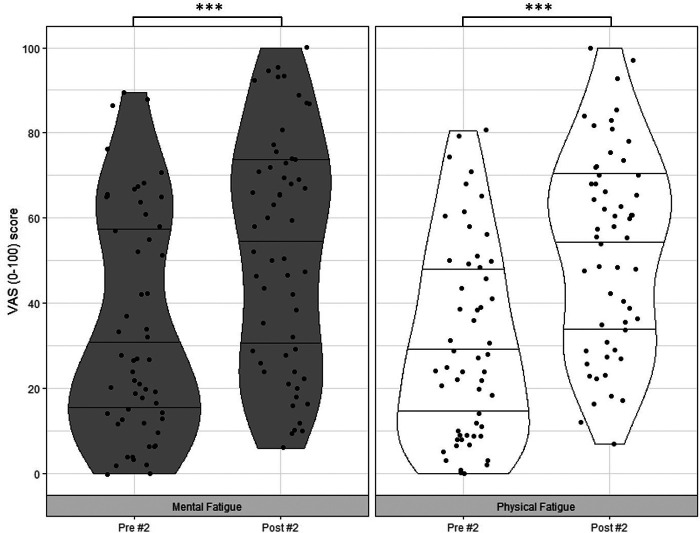
Violin Plots including data points for pre- and post-competition ratings of *mental fatigue* and *physical fatigue* for competition #2: minimum, Q1, median Q4 and maximum scores. Notes: VAS = Visual Analogue Scale. ***p < .001.

Regarding the competition duration, results show that the net duration of #1 was 63.20 ± 15.65 min in total and #2 lasted 60.79 ± 11.45 min in total. Both *mental fatigue* and *physical fatigue* significantly changed between T1 and T2 (*mental fatigue*: *z* = −4.59, *p* < .001, *r* = .61; *physical fatigue*: *z* = −4.38, *p* < .001, *r* = .58) and between T3 and T4 (*mental fatigue*: *z* = −3.93, *p* < .001, *r* = .52; *physical fatigue*: *z* = −4.63, *p* < .001, *r* = .62) (Figure 2, Figure 3).

Differences between pre- and post-competition ratings of *mental fatigue* and *physical fatigue*, acute changes in *mental fatigue* comparative to *physical fatigue*, and between-competition differences in pre-ratings of *mental fatigue* and *physical fatigue* are shown in Table 1.

**Table 1:** Wilcoxon signed rank statistics for MF and PF comparisons.

*Mental fatigue & physical fatigue comparisons*	Mean ± SD	*p*-Value	*r*
*Mental fatigue* T1 *Mental fatigue* T2	25.13 ± 21.39 43.40 ± 26.06	< .001	.61
*Physical fatigue* T1 *Physical fatigue* T2	23.71 ± 21.75 42.23 ± 24.33	< .001	.58
*Mental fatigue* T3 *Mental fatigue* T4	34.26 ± 26.31 52.62 ± 27.46	< .001	.52
*Physical fatigue* T3 *Physical fatigue* T4	30.53 ± 22.84 52.84 ± 23.50	< .001	.62
*Mental fatigue* (T2 minus T1) *Physical fatigue* (T2 minus T1)	18.27 ± 26.92 18.62 ± 27.05	.829	.29
*Mental fatigue* (T4 minus T3) *Physical fatigue* (T4 minus T3)	18.36 ± 31.36 22.31 ± 28.72	.285	.14

Relating to #1, shared variance between change in *mental fatigue* and *physical fatigue (T2 minus T1)* significantly differs (*r* = .28, *p* < .05). Thus, the proportion of shared variance between change in *mental fatigue* and *physical fatigue* as two ranked variables was 7.9%. For competition bout #2, results showed that shared variance between change in *mental fatigue* and *physical fatigue (T4 minus T3)* also significantly differs (*r* = .43, *p* < .01). The proportion of shared variance between change in *mental fatigue* and *physical fatigue* as two ranked variables was 18.6%.

Regarding *total shooting score's* intercept-only model results, a significant grand-mean of *total shooting score* of 605.95 points was indicated (*p *< .001, 95% CI [602.28, 609.22]). ICC calculation revealed that 70.6% of the *total shooting score* variation occurred across participants. Results of the final models for the *total shooting score* included random intercepts and fixed slopes for predictor variables measurement points and intervention groups revealed no significant effect (*p* > .05) of intervention groups, measurement points nor interactions between intervention groups and measurement points.

**Figure 4:**
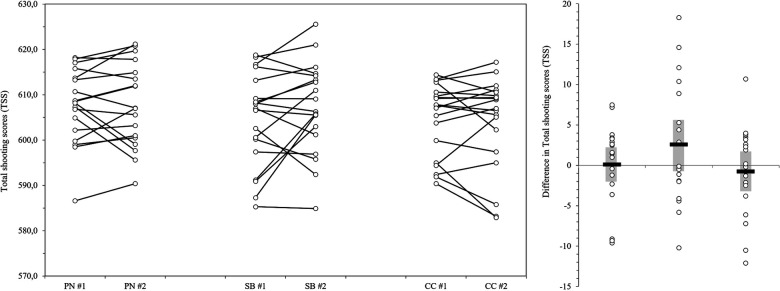
Individual changes in *total shooting scores* and differences in *total shooting scores* T1 = pre-competition 1, T2 = post-competition 1, T3 = pre-competition 2, T4 = post-competition 2, PN = powernap, SB = systematic breathing, CC = control condition, TSS = total shooting scores, circles = individual TSS/individual difference in TSS, black bars = mean of the difference in TSS, grey sector = 95% CI.

On a descriptive level, results of shooting performance showed an average *total shooting score* for PN (#1 = 607.6 ± 8.23, #2 = 607.7 ± 9.28), SB (#1 = 604.5 ± 10.39, #2 = 607.1 ± 10.04), and CC (#1 = 604.7 ± 8.09, #2 603.9 ± 10.34). Individual changes of the *total shooting score* from #1 to #2 as well as differences in the *total shooting score* separated by intervention groups are displayed in Figure 4. Results revealed an average increase of the *total shooting score* for PN of 0.1 ± 4.65, 95% CI [2.25, −2.05], an increase for SB of 2.58 ± 6.96, 95% CI [5.72, −0.55] and a decrease for CC of −0.76 ± 5.1, 95% CI [1.54, −3.05]. On an individual level, statistically relevant increases of shooting performance by using powernap or systematic breathing could be found.

**Figure 5:**
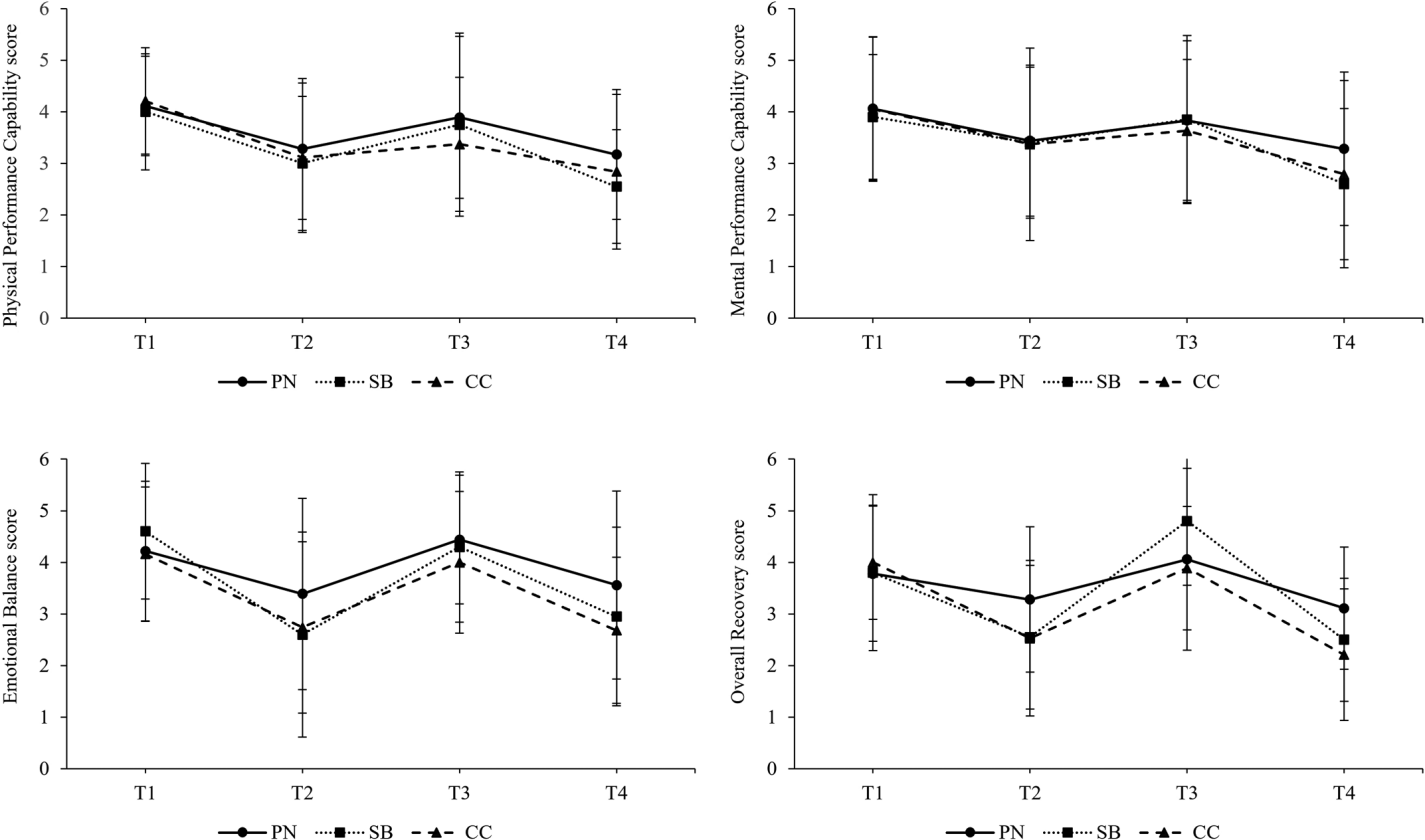
Development of recovery specific items of the SRSS over the course of a simulated competition day. T1 = pre-competition 1, T2 = post-competition 1, T3 = pre-competition 2, T4 = post-competition 2, PN = powernap, SB = systematic breathing, CC = control condition.

**Figure 6:**
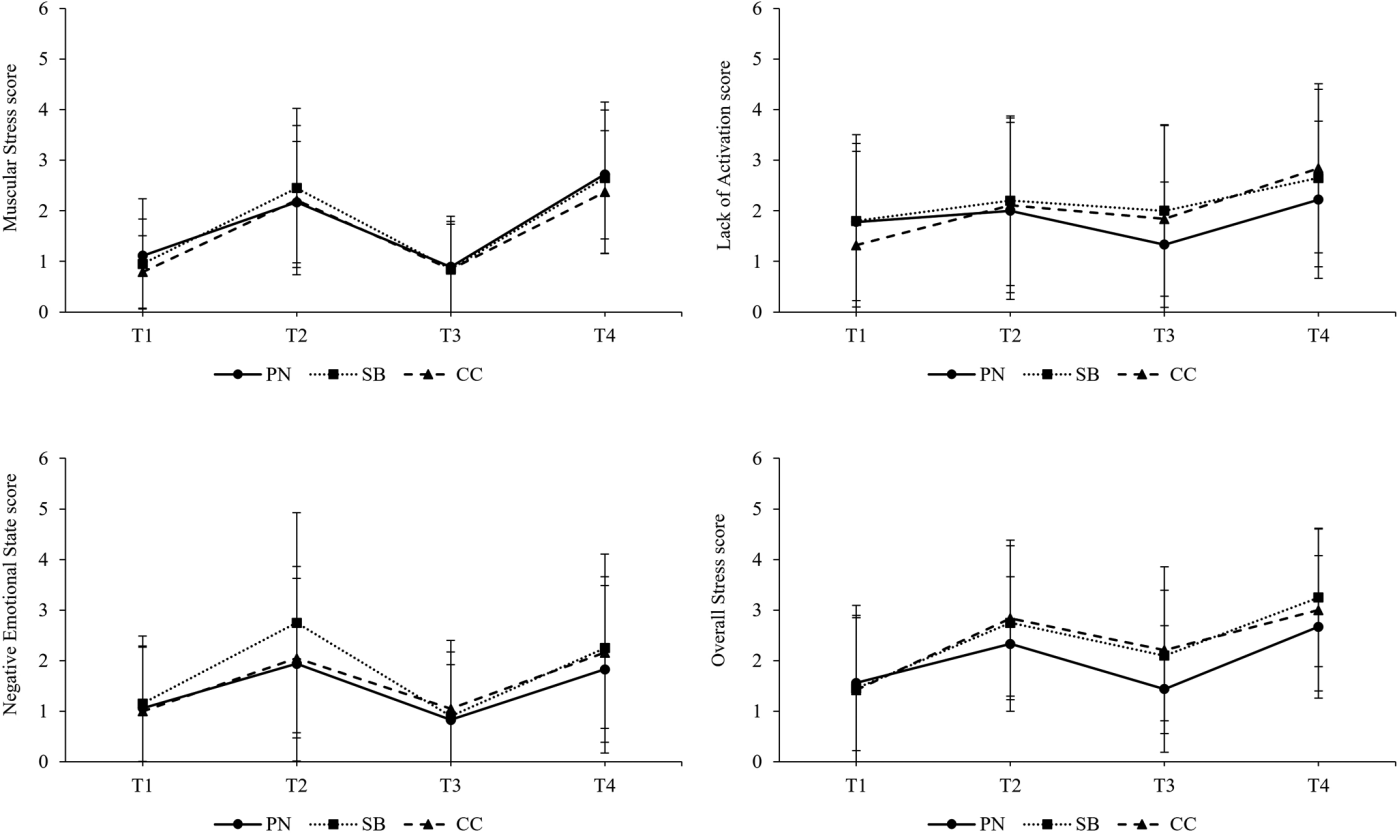
Development of stress specific items of the SRSS over the course of a simulated competition day. T1 = pre-competition 1, T2 = post-competition 1, T3 = pre-competition 2, T4 = post-competition 2, PN = powernap, SB = systematic breathing, CC = control condition. Results presented as mean ± SD.

The following section only presents significant results of the final models for SRSS scores, for values of *mental fatigue*, *physical fatigue*, *concentration,* and *motivation* as well as RPE scores including interaction of predictors measurement point intervention group. The intercept-only model for *Physical Performance Capability* revealed a significant grand-mean of 3.39 (*p* < .001, 95% CI [3.06, 3.72]) and ICC calculations indicated that 22.2% of variation in *Physical Performance Capability* scores occurred across participants. Intercept-only model's grand-mean of *Mental Performance Capability* was statistically significant and amounted to 3.48 (*p* < .001, 95% CI [3.08, 3.89]). The calculated ICC was 29.4%. The intercept-only model for *Emotional Balance* showed a significant grand-mean of 3.63 (*p* < .001, 95% CI [3.33, 3.92]) and an ICC of 7.0%. The grand mean of *Overall Recovery* provided by the intercept-only model was statistically significant and amounted to 3.34 (*p* < .001, 95% CI [3.04, 3.63]). ICC calculation indicated that 11.4% of variation occurred across participants. Intercept-only model's grand-mean for *Muscular Stress* was statistically significant and amounted to 1.68 (*p* < .001, 95% CI [1.39, 1.97]), the calculated ICC was 14.3%. For *Lack of Activation*, intercept-only model results also showed a significant grand-mean which amounted to 2.02 (*p* < .001, 95% CI [1.62, 2.42]). ICC calculations indicated that a 20.3% variation of *Lack of Variation* occurred across participants. For scores of *Negative Emotional State*, the intercept-only model indicated a statistically significant grand-mean of 1.61 (*p* < .001, 95% CI [0.40, 1.04]). ICC calculation results indicated an amount of 16.4% variation across participants. *Overall Stress* scores' significant grand-mean indicated by the intercept-only model amounted to 2.31 (*p* < .001, 95% CI [0.46, 1.06]). The variation across participants (ICC) caused a 20.8% variation in *Overall Stress* scores. Regarding models including predictors, no significant effects of intervention group or interactions of measurement points and intervention groups on SRSS scores were found, except for *Overall Recovery*. The model included random intercepts and fixed slopes for measurement points, intervention groups and interactions of measurement points and intervention groups as predictors. Results indicated a significant group-unspecific intercept for *Overall Recovery* of 3.83 (*p* < .001, 95% CI [3.25, 4.41]) and a significant increase of 1.33 in *Overall Recovery* on average when SB was applied (*p* < .05, 95% CI [0.16, 2.49]). The development of SRSS scores separated by intervention groups are illustrated in Figure 5 and Figure 6.

**Figure 7:**
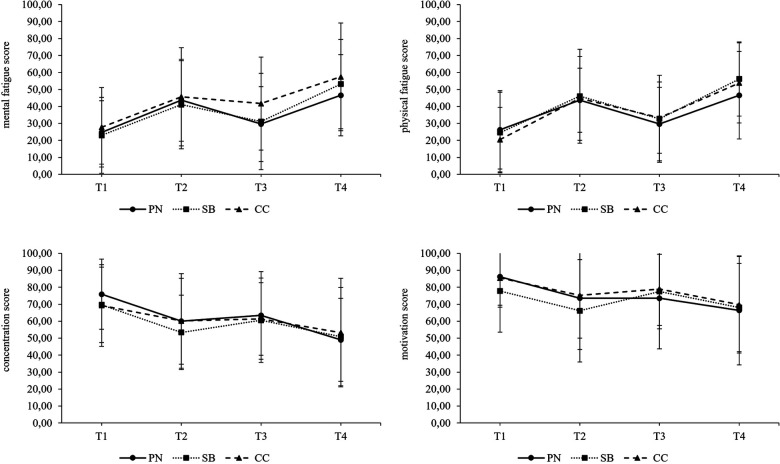
Development of fatigue scores over the course of a simulated competition day. T1 = pre-competition 1, T2 = post-competition 1, T3 = pre-competition 2, T4 = post-competition 2, PN = powernap, SB = systematic breathing, CC = control condition. Results presented as mean ± SD.

The intercept-only model for *mental fatigue* revealed a significant grand-mean of 40.34 (*p* < .001, 95% CI [33.80, 46.92]) and ICC calculation indicated that 21.7% of variation in ratings of *mental fatigue* scores occurred across participants. The intercept-only model's grand-mean of *physical fatigue* scores was statistically significant and amounted to 38.96 (*p* < .001, 95% CI [32.82, 45.16]). The calculated ICC was 21.20%. The intercept-only model for *concentration* showed a significant grand-mean of 59.69 (*p* < .001, 95% CI [52.34, 67.01]) and an ICC of 34.9%. The intercept-only model for *motivation* provided a significant grand-mean of 74.30 (*p* < .001, 95% CI [66.31, 82.25]). ICC calculation indicated that 46.1% of variation occurred across participants. Models including the predictor variables *measurement points* and *intervention group* (respectively their interaction) revealed no statistically significant effect of the selected predictor variables. The development of the scores of *mental fatigue*, *physical fatigue*, *concentration,* and *motivation* separated by intervention groups are illustrated in Figure 7.

**Figure 8:**
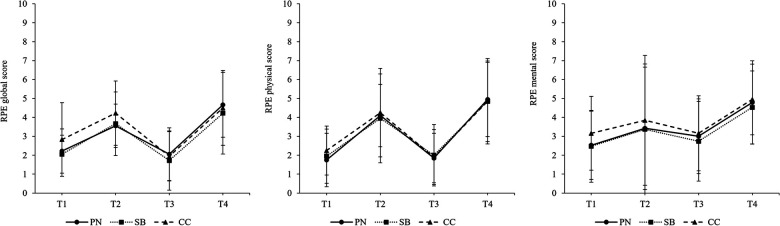
Development of differential RPE scores over the course of a simulated competition day. T1 = pre-competition 1, T2 = post-competition 1, T3 = pre-competition 2, T4 = post-competition 2, PN = powernap, SB = systematic breathing, CC = control condition. Results presented as mean ± SD.

The intercept-only model for *RPE global* revealed a significant grand-mean of 3.34 (*p* < .001, 95% CI [2.96, 3.72]) and ICC calculation indicated that 10.2% of variation in ratings of *RPE global* occurred across participants. The intercept-only model for *RPE physical* showed a significant grand-mean of 3.23 (*p* < .001, 95% CI [2.79, 3.67]) and an ICC of 14.1%. The intercept-only model for *RPE mental* provided a significant grand-mean of 3.58 (*p* < .001, 95% CI [3.20, 3.97]). ICC calculation indicated that 8.9% of variation occurred across participants. Models including the predictor variables *measurement points* and *intervention group* (respectively their interaction) revealed no statistically significant effect of selected predictor variables on RPE scores. The development of RPE scores separated by intervention groups are illustrated in Figure 8.

### Evaluation of the recovery break

Relating to the efficacy of the implemented interventions, the descriptive data of the manipulation check of recovery interventions obtained higher scores for PN (4.59 ± 1.42) and SB (4.68 ± 0.82) compared to CC (3.05 ± 1.43). Similar results were found for the appreciation with higher scores for PN (4.88 ± 1.45) and SB (4.79 ± 0.98) compared to CC (2.35 ± 1.50). The assessment of feasibility stays on a similar level for all three interventions (PN = 3.94 ± 1.85; SB = 4.16 ± 1.21; CC = 4.05 ± 1.50).

## Discussion

The aim of the present study was firstly to assess the perception and the change of mental and physical fatigue in air rifle athletes over the course of a simulated competition day and secondly to examine acute effects of mental recovery strategies on recovery-stress states, subjective fatigue states, perceived exertion, and shooting performance during an implemented mental recovery break.

Regarding the holistic approach of Van Cutsem et al. (13), to strengthen the understanding of how mental fatigue impairs human performance, in the present study we focus on subjective and behavioural parameters of mental fatigue and mental recovery. Due to unchangeable framework conditions, no additional peripheral neurophysiological parameters could be included in this study. In order to pursue these aims, the basic idea of this study was to choose the best possible applied setting. Therefore, with regard to the results, it has to be emphasized that although competition-like situations (internal and external) could be created over all test days, these conditions could not come close to the real demands of the athletes (e.g., pressure loads, tension, atmosphere). On the continuum of laboratory and applied research, conducting simulated competition days is a first and important but not yet sufficient step.

Results show that during #1 as well as during #2 subjective measures of both *mental fatigue* and *physical fatigue* significantly increased. However, changes in each of these measures were not strongly related. Agreeing with the results of Russell et al. (30), the present data underline that *mental fatigue* and *physical fatigue* are largely different constructs. Analysis found a low proportion of variance of change in *mental fatigue* to be explained by variance in *physical fatigue*, with 81.9% (#1) or 76.8% (#2). The mean pre-post competition changes and post-competition ratings of *mental fatigue* (#1 = 43.4 ± 26.06; #2 = 52.62 ± 27.46) found in the present study are similar to those reported by Russell et al. (30) in elite female netballers following a match over four quarters of 15 min (44.73 ± 24.47). Moreover, the post-competition ratings of *mental fatigue* are in line with the results revealed by Veness et al. (10) in elite male cricketers following 35 min of exposure to the incongruent Stroop Test (46.6 ± 5.86), Smith et al. (39) in male soccer players who performed 30 min modified Stroop Task (58 ± 22), and Kosack et al. (38) in national elite male badminton players who conducted a 60 min inhibitory Stroop Task (57 ± 23), who all used computer-based treatments to induce *mental fatigue*. In contrast to different high-intensity intermittent sports (e.g., football, netball, badminton), the requirements of air rifle shooting consist to a great proportion of high cognitive efforts (e.g., concentration capacity, attentional focus, impulse control) leading to an increased perception of *mental fatigue*, but results also show higher ratings of *physical fatigue* post-competition. The post-competition ratings (T2, T3) of *physical fatigue* (#1 = 42.32 ± 24.33; #2 = 52.84 ± 23.51) are also similar to the results of Russell et al. (30). Thus, the demands of the simulated competition day (i.e., two competition bouts over a period of a maximum of 75 min) conducted in the present study appear to be not only one-sided (e.g., primarily cognitive stressors) but also multidimensional (e.g., cognitive, physical, and emotional stressors) leading to an increase of *mental fatigue* as well as an increased *physical fatigue* in the air rifle athletes. In addition, the development of the SRSS items and the differential RPE scores from T1 to T2 or T3 to T4 respectively (e.g., increase in *Overall Stress*, *Muscular Stress*, *Negative Emotional State*, *RPE general*, *RPE physical*, *RPE mental*; decrease in *Overall Recovery* and *Emotional Balance*) confirm this statement.

Relating to the acute change (T2 minus T1, T4 minus T3) in *mental fatigue* #1 (18.27 ± 26.92) and #2 (18.36 ± 31.36), results reflect the effects of the mental demands which are experienced by the air rifle athletes for a sustained duration (i.e., competition day with two competition bouts) resulting in an acute state of *mental fatigue* (3, 4, 10). Moreover, the additional increase of *mental fatigue* in #2 give indications regarding a more cumulative aspect of *mental fatigue*, which means that the perception of *mental fatigue* accumulates in repetitive acute bouts of mental demands. Athletes' perception of mental fatigue across training, preparation and competition periods appears to be individual, but findings of Russell et al. (2021) indicates that a sustained exposure of demands of sports competitions as well as repeated stimulus of cognitive demands over an extended period can lead to an increase of susceptibility perceiving mental fatigue. Therefore, further studies aiming at the monitoring of *mental fatigue* over a longer duration in an applied setting may improve the understanding of *mental fatigue* in the elite sport setting (14). In addition, similar variations were found for the acute change in *physical fatigue* #1 (18.62 ± 27.05) and #2 (22.31 ± 28.72).

Focusing on the second aim of the study, results revealed no significant group-based acute effects of the use of powernap and systematic breathing on shooting performance in simulated air rifle competition. On an individual level, however, statistically relevant improvements of *total shooting score* from #1 to #2 could be found. In air rifle shooting a minimal change (e.g., improvement, deterioration) in the shooting score in a single bout can be crucial (e.g., qualification for the final). Taken into account, that no familiarization sessions for the mental recovery strategies could be implemented within the study design and that literature underlines that these mental recovery strategies have to be practiced regularly in order to be effective in training and competition (17, 43), significant acute effects of a single application of mental recovery strategies during the course of the study could not be expected. In addition, it has to be underlined, that the application of mental recovery strategies is highly individual, so that not every athlete could apply the most suitable and effective strategy for himself or herself. This is also shown in the shooting results illustrating both a significant increase and a decrease in shooting performance by using powernaps and systematic breathing. On this basis, recommendations for individual appropriate mental recovery strategies should be derived for the athlete in a follow-up to this study. Regarding acute effects of mental recovery strategies on psychological and perceptual measures, results revealed a significant increase of *Overall Recovery* when using systematic breathing in the recovery break before the start of #2. This positive change could be explained with the fact, that breathing is the basis of a wide range of relaxation strategies and the use of SB was therefore easy to implement (43). Moreover, breathing regulation plays an important role in air-rifle shooting and therefore the participants were more familiar with the use of SB compared to the use of a powernap. No further significant acute effects on psychological and perceptual measure could be found. One reason for this could be that recovery is a highly individual process and recovery strategies have to match an individual's specific needs (43, 45). Optimal short-term recovery can only be achieved when recovery activities are consciously planned according to situational and environmental needs (46). Thus, focusing on rest periods, consciously used (pro-active) mental recovery strategies appear to be beneficial to re-establish essential resources and to counteract mainly subjective facets of mental fatigue in order to maintain performance readiness on a physical, mental, and emotional level (9). A second reason could be that the participants even perceived the control condition as recovering on a mental and emotional level, which leads to a similar perception of fatigue and recovery-stress states. This could be due to the fact that the control condition comprised moderate mental demands without providing systematic guidance compared to the implemented mental recovery strategies. Even if the participating athletes rarely use certain recovery strategies in training and competition, an additional factor can be that these participants have learned from experience ways of recovery that they (subconsciously) used in the control group. A third reason could be that the applied mental recovery strategies (i.e., powernap, systematic breathing) should be learned and practiced regularly before they can be applied efficiently in training and competition (17). Hence, significant differences between the respective mental recovery intervention and CC can potentially be expected after a sufficiently long practice phase. Due to some organizational and temporal framework conditions on site (e.g., training camps, selection tournaments, upcoming competition highlights), a practice phase for a sufficient number of athletes could not be realized in the run-up to the study. This should be taken into account for future studies.

However, changes revealed that an implemented recovery break, independent of the specific recovery strategy, appears to have a general recovery-promoting effect and may be beneficial to participants' perceptions of their subjective fatigue states and recovery-stress states. Since rest periods primarily aim at a re-establishment of pre-performance states as well as personal resources (46, 47) and mental recovery includes both mental and emotional aspects of recovery (48), results illustrate that a rest period with any of the mental recovery strategies leads to improvements of *Overall Recovery*, *Emotional Balance*, and the perception of *Concentration* as well as to reductions of *Negative Emotional State*, *mental fatigue* and *RPE* scores in response to competition #1. However, the use of a second control condition (i.e., removal of a recovery break) seems warranted for further information and to test this assumption.

Results suggest that a mental break may buffer mental fatigue outcomes by increasing mental and emotional recovery states with the primary association of mental fatigue manifestations with subjective and behavioural markers such as feelings of tiredness, lack of energy, and perception of increased effort (15, 49). Thus, when matching individual needs, a mental break between competition bouts can be beneficial to cope with mental demands of competition and to promote a state of feeling mentally recovered with corresponding feelings of being physically and mentally recovered, concentrated, receptive, alert, and balanced (23, 34). In this respect, the assessment of individual appropriate mental recovery strategies and a regular use appeared to be more beneficial. Moreover, this is consistent with the idea of recovery self-regulation comprising the active transformation of a restraining state (e.g., mental fatigue) to a beneficial state (e.g., optimal state of recovery) (1). Therefore, further studies in an applied sport setting are necessary to gain new insights on effects of mental breaks on competition days.

Despite the strengths of the study, some potential limitations have to be considered and amended by future research. First, the study sample was rather small, resulting from the difficulty to get the participants through the rigorous study design with three separated test days (e.g., dropout, invitations to training camps, illness). Therefore, some of the non-significant effects reported in the present study might have been significant with a larger sample of participants with more statistical power. Furthermore, the reduced skill level of the elite development athletes has to be considered. Future studies should also examine a sample of elite air rifle athletes with a greater experience in training and competition bouts. Second, the present study tries to represent a rather realistic competition environment (i.e., simulated competition bouts), future research however should transfer the present study design to an applied setting including, for example, real competition settings, real opponents, and real high-pressure experiences. Third, to improve the metacognition of mental fatigue, physical fatigue, concentration and motivation, short definitions were provided. Regarding the characteristics of the participants (e.g, age, pre-knowledge), future research should use target group-specific, unambiguous, and more straightforward definitions to ensure an even clearer understanding of and a distinction between these constructs. Fourth, due to some organizational challenges, no familiarization session for the applied recovery strategies could be performed in advance or could be integrated into the rigorous study design. The implementation of a training session can be a useful goal for future research so that the participants can already deal with recovery strategies to be examined. Fifth, it should be noted that the present study only focused on the collection of multiple subjective measures to show acute effects on the perception of recovery-stress states and fatigue states. With regard to the sport-specific determinants of air rifle shooting, further studies are required with an additional focus on physiological (e.g., HR, HRV), hormonal (e.g., cortisol) and respiratory measures to get further information on the perception of mental fatigue and the psychophysiological responses of mental recovery strategies. Sixth, as already mentioned, the choice of magazines as an appropriate CC needs to be discussed for the use in further studies investigating effects of mental recovery strategies.

## Conclusion

In conclusion, the present study found increases in *mental fatigue* and *physical fatigue* during a simulated air rifle competition day including two competition bouts with development air rifle athletes. Moreover, study results support the assumption that *mental fatigue* is a largely separate construct to *physical fatigue* (30) and should therefore also be considered. As a novel aspect, the study examined acute effects of mental recovery strategies in air-rifle shooting as a countermeasure to mental fatigue. Results revealed no significant group-based acute effects of the use of mental recovery strategies on shooting performance as well as psychological and perceptual measures. On an individual level, however, isolated cases could be found showing statistical relevant increases of shooting performance by using powernap or systematic breathing. Overall, a key experience of recovery involves a reduction in mental demands of competition and therefore recovery following competitions does not just involve recovering physically, it also involves recovering mentally (31). The use of strategies to accelerate mental recovery (e.g., powernap, systematic breathing) can be a first step to manage a state of mental fatigue, but further studies in an applied setting of different sports with the focus on characteristics of multisport events as well as acute and cumulative effects of *mental fatigue* are needed.

## Data Availability

The raw data supporting the conclusions of this article will be made available by the authors, without undue reservation.
